# Complete migration of a composite mesh into small bowel incidentally found during laparotomy for colectomy in an asymptomatic patient: a case report

**DOI:** 10.1186/s13256-020-02540-4

**Published:** 2020-10-31

**Authors:** Pramodh Chandrasinghe, Asantha De Silva, Ayomi Welivita, Kemal Deen

**Affiliations:** 1 Department of Surgery, Faculty of Medicine, University of Kelaniya, Ragama, Sri Lanka; 2 Department of Obstetrics and Gynecology, Faculty of Medicine, University of Kelaniya, Ragama, Sri Lanka; 3 Kings Hospital, Colombo 5, Sri Lanka

**Keywords:** Mesh migration, Composite mesh, Case report, Mesh complications

## Abstract

**Background:**

Composite meshes are used for incisional hernia repair because they enable intraperitoneal mesh placement due to their dorsal surface, which is made of inert material. We report, for the first time, to our knowledge, a case of composite mesh migration detected incidentally during a laparotomy for colon cancer in an asymptomatic patient.

**Case presentation:**

Our patient was a 71-year-old South Asian man who underwent ventral mesh repair following a postoperative complication after right hemicolectomy for colon cancer. The patient was diagnosed with a metachronous sigmoid cancer 5 years later, for which he underwent laparotomy. During laparotomy, a migrated mesh was incidentally found and extracted from his proximal ileum without any evidence of abscess or fistula formation.

**Conclusion:**

To our knowledge, this is the first report of an incidentally found migrated composite mesh from a bowel lumen in an asymptomatic patient.

## Background

Use of mesh has become standard in a majority of incisional hernia repair to achieve a tension-free repair and reduce recurrence [1]. The composite mesh is designed to reduce the risk of bowel adhering to the mesh by including an absorbable layer to one surface, enabling intraperitoneal mesh placement [2]. It is supposed to reduce complications such as mesh erosion into bowel caused by the use of intraperitoneal polypropylene-only meshes. Reports of mesh migration, both composite and nonabsorbable types, are found in the literature, although they are always associated with apparent clinical signs and symptoms such as abscess formation, enterocutaneous fistula formation, or bowel obstruction [3, 4]. We report a case of a composite mesh completely migrating into the small bowel found during laparotomy for an unrelated issue.

## Case presentation

**Fig. 1:**
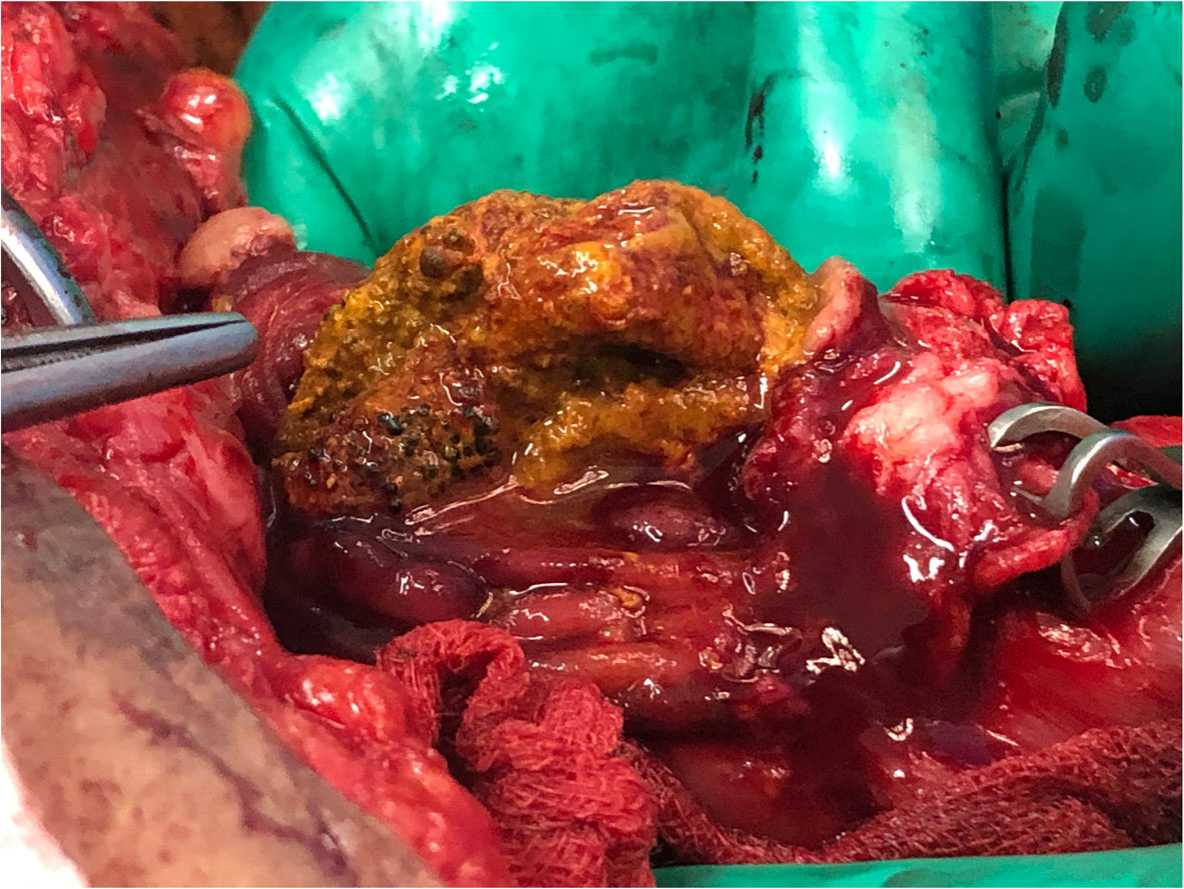
The migrated mesh inside the proximal ileum seen through the enterotomy

**Fig. 2:**
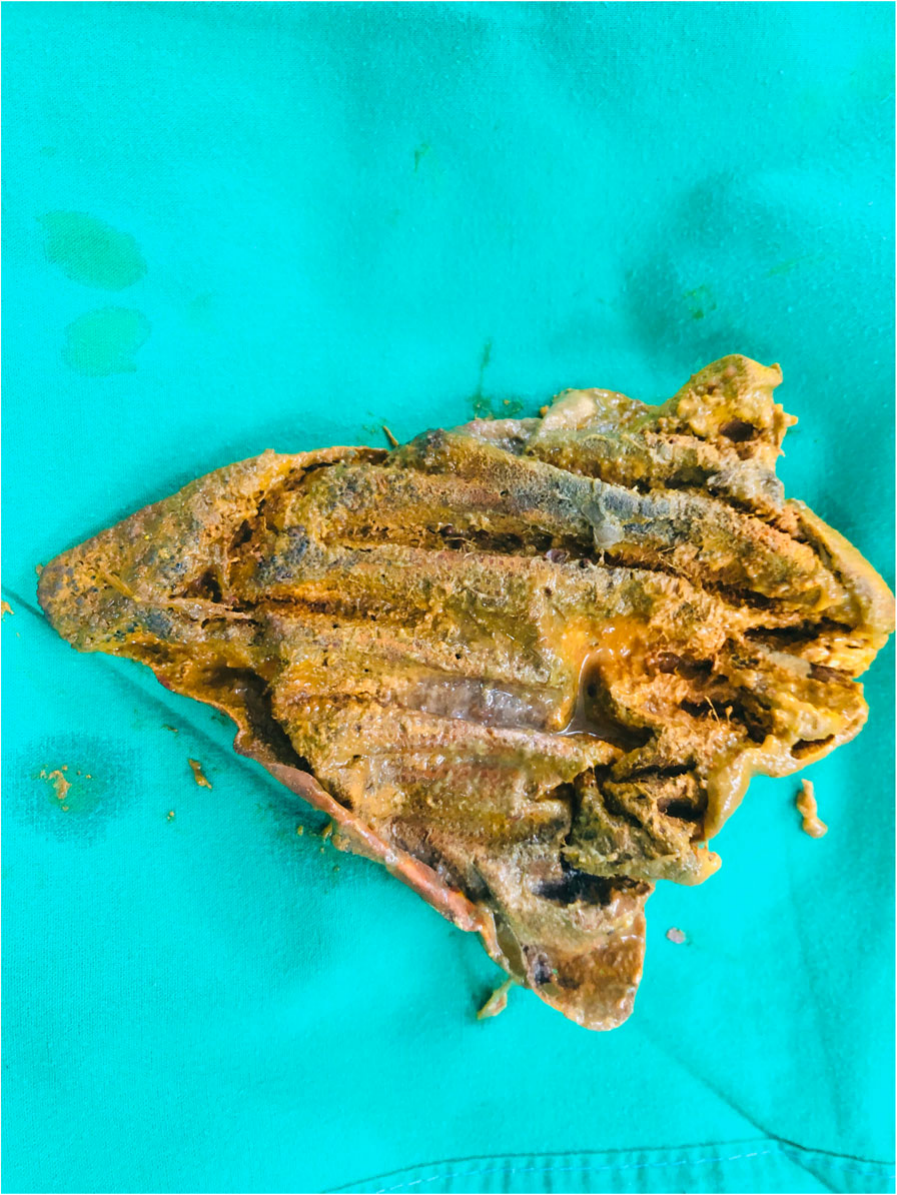
The extracted mesh with heavy deposition of fecal matter

A 71-year-old South Asian man who had under gone a right hemicolectomy for an adenocarcinoma of the colon was found to have a metachronous cancer in the sigmoid colon during surveillance colonoscopy. Following his primary surgery at another institution, he has developed an anastomotic leak during the immediate postoperative period, for which a second laparotomy had been performed on day 5 with a revision of the anastomosis. During the first postoperative week, he had developed an enterocutaneous fistula with primary wound failure. His case was then taken over by the authors for the management of the fistula, which was done nonoperatively. Spontaneous healing of the fistula took 10 months, after which the patient underwent an abdominal wall reconstruction with an inlay polypropylene/polyglactin 910 composite mesh. The reconstruction was done 16 months after the primary surgery, and the mesh was fixed using polypropylene sutures. The patient remained asymptomatic until he was diagnosed with a sigmoid cancer on the basis of surveillance colonoscopy 5 years after the primary event. Following histological confirmation and staging with contrast-enhanced computed tomography, he was scheduled for laparotomy with subtotal colectomy and ileorectal anastomosis. During surgery, several loops of bowel were found to be tightly adherent to the anterior abdominal wall. On palpation, a hard, elongated mass was felt inside one of the bowel loops in the proximal ileum. After separating the loops from the posterior rectus sheath, an enterotomy was made to find the mesh inside the bowel lumen (Fig. 1). The mesh was not adherent to the bowel wall and could be extracted without difficulty. It was heavily deposited with fecal matter, indicating that it had been in the bowel lumen for a long period (Fig. 2). There was no evidence of an abscess, fistula, or sinus formation in the surrounding area. The enterotomy was closed with a side-to-side stapler anastomosis, and the patient underwent subtotal colectomy with ileorectal anastomosis with an uneventful recovery.

## Discussion

There have been few cases of composite mesh migration reported in the literature, although the patients have been symptomatic in every instance, with a complication such as enterocutaneous fistula, bowel obstruction, or intra-abdominal mass formation [3, 5, 6]. To our knowledge, this is the first report of a composite mesh migration without causing symptoms or signs. Herrera *et al*. reported the first case of mesh migration following incisional hernia repair [7]. A foreign body laying inside the peritoneal cavity has the tendency to migrate into bowel or through the skin into the exterior. Composite meshes were designed to eliminate this by having a more porous nonabsorbable surface facing anteriorly to allow ingrowth of connective tissue while the inert surface made of an absorbable material faces inside, preventing adhesions [2]. The migration process may occur either acutely due to a postoperative inflammatory process or as a slow process [8]. It is possible that the initial event in our patient was the dislodgment of the mesh from the anchoring sutures. Several small bowel loops could have adhered to the exposed polypropylene surface, and slow erosion into a loop would have started. The initial necrosis of the bowel wall due to the inflammation would have been sealed off by the surrounding bowel loops, preventing an abscess formation. Once the mesh was slowly internalized, it is possible that the opening would simultaneously have been sealed off by the bowel loops; hence, the bowel mass detected during this laparotomy would have formed. Similar silent erosion by a retained surgical sponge has been reported [9]. A slow process is likely to cause foreign body migration without leakage of content, but, given the size of the extracted mesh, it is highly likely to cause a fistulous tract. Soler *et al.* reported one case of composite mesh migration into the small bowel under experimental conditions in a murine study [10]. Mesh migration to different parts of the intestine in humans has been reported [3, 7, 11, 12]. In all cases, the patients were symptomatic due to sepsis or obstruction. The uniqueness of our patient’s case is that at no stage during the preceding 4 years had the patient experienced such a complication. The staging computed tomography (CT) performed in this patient did not reveal the presence of a mesh in the small bowel. The literature also indicates similar failures of CT scans to detect the presence of a mesh inside the bowel [3].

## Conclusion

Incisional hernia repair involves the use of novel mesh devices designed to reduce complications. Composite meshes are designed to prevent bowel adhesion by using an inert material to the dorsal surface, although several cases of mesh migration have been reported. Reports of composite mesh migration are all associated with patients presenting with either infective or obstructive symptoms. This case report, for the first time, to our knowledge, raises the possibility of complete migration of a composite mesh without causing symptoms or signs.

## Data Availability

Available on reasonable request.

## Abbreviations

CT: Computed tomography
